# Gadolinium-enhanced cardiac magnetic resonance imaging: administered dose in relationship to United States Food and Drug Administration (FDA) guidelines

**DOI:** 10.1186/1532-429X-14-S1-P46

**Published:** 2012-02-01

**Authors:** Marcelo Nacif, Andrew E Arai, Joao A Lima, David A Bluemke

**Affiliations:** 1 National Institutes of Health, Radiology and Imaging Science, Bethesda, MD, USA; 2 Division of Cardiology, Johns Hopkins University School of Medicine, Baltimore, MD, USA; 3 Cardiovascular and Pulmonary Branch, National Heart, Lung, and Blood Institute, National Institutes of Health, Bethesda, MD, USA; 4 Molecular Biomedical Imaging Laboratory, National Institute of Biomedical Imaging and Bioengineering, Bethesda, MD, USA

## Summary

The Food and Drug Administration (FDA) has recently recommended that the label dose of Gadolinium (Gd)-based contrast agents (GBCA) not be exceeded. The primary concern for high dose GBCAs application is nephrogenic systemic fibrosis (NSF). For the majority of conventional GBCA, the label dose is 0.1 mmol/kg. GBCA use for evaluation of myocardial disease is currently considered off-label use in the United States. Little information is present in the literature regarding optimal GBCA dose for CMR. Therefore, we evaluated the current status of GBCA use for CMR of the myocardium as presented in the peer-reviewed literature, emphasizing trends before and after nephrogenic fibrosis guidelines were issued in 2008. This meta-analysis showed that the median GBCA dose for English peer reviewed publications on CMR (19,934 patients) was 0.2 mmol/kg. Further, no change in mean or median gadolinium dose was present before, versus after the FDA issued GBCA black box warnings (p>0.05). This appears surprising, given the amount of attention and many publications regarding NSF. It remains to be seen if future CMR studies will incorporate and report lower gadolinium doses. Clinical trials should be supported to determine the appropriate doses of gadolinium enhancement of the myocardium.

## Background

Myocardial delayed enhancement MRI was originally validated using higher than label-recommended doses of gadolinium chelate. The objective of this study was to evaluate available evidence for various gadolinium dosing regimens used for cardiac MRI. The relationship of gadolinium dose warnings (due to nephrogenic systemic fibrosis) announced in 2008 to gadolinium dosing regimens was also examined.

## Methods

We conducted a meta-analysis of peer reviewed publications from January, 2004 to December, 2010. Major subject search headings (MeSh) terms from the National Library of Medicine’s PubMed were: contrast media, gadolinium, heart, magnetic resonance imaging; searches were limited to human studies with abstracts published in English. Case reports, review articles, editorials, MRA related papers and all reports that did not indicate gadolinium type or weight-based dose were excluded. For all included references, full text was available to determine the total administered gadolinium dose on a per kg basis. Average and median dose values were weighted by the number of subjects in each study.

## Results

399 publications were identified in PubMed; 233 studies matched the inclusion criteria, encompassing 19,934 patients with mean age 54.2±11.4 (range 9.3 to 76 years). 34 trials were related to perfusion testing and 199 to myocardial delayed enhancement. In 2004, the weighted-median and weighted-mean contrast dose were 0.15 and 0.16±0.06 mmol/kg, respectively. Median contrast doses for 2005-2010 were: 0.2 mmol/kg for all years, respectively. Mean contrast doses for the years 2005-2010 were: 0.19±0.03, 0.18±0.04, 0.18±0.10, 0.18±0.03, 0.18±0.04 and 0.18±0.04 mmol/kg, respectively (p for trend, NS). Gadopentetate dimeglumine was the most frequent gadolinium type [114 (48.9%) studies]. No change in mean gadolinium dose was present before, versus after the FDA black box warning (p>0.05). Three multi-center dose ranging trials have been published for cardiac MRI applications.

## Conclusions

Cardiac MRI studies in the peer-reviewed published literature routinely use higher gadolinium doses than FDA indicated dose. Clinical trials should be supported to determine the appropriate doses of gadolinium enhancement of the myocardium.

## Funding

This study was supported by the NIH intramural research program.

**Table 1:** Characteristics of publications included in the meta-analysis

Year	Studies	Participants	Age (years)	Perfusion studies	LGE studies	Median GBCA dose (mmol/Kg)*	Mean GBCA dose (mmol/Kg)*
2004	22 (9.5)	697 (3.5)	56.1±8.7	6 (17.6)	16 (8.1)	0.15	0.16±0.06
2005	19 (8.2)	2,123 (10.7)	54.4±8.4	2 (5.9)	17 (8.6)	0.2	0.19±0.03
2006	26 (11.1)	4,366 (22.0)	54.9±11.3	5 (14.8)	21 (10.5)	0.2	0.18±0.04
2007	31 (13.3)	1,123 (5.6)	46.1±16.9	3 (8.9)	28 (14.1)	0.2	0.18±0.04
2008	40 (17.1)	2,264 (11.3)	55±8.9	7 (20.5)	33 (16.5)	0.2	0.18±0.03
2009	45 (19.3)	3,965 (19.9)	56.2±10.3	5 (14.7)	40 (20.1)	0.2	0.18±0.04
2010	50 (21.5)	5,396 (27.0)	55±11.3	6 (17.6)	44 (22.1)	0.2	0.18±0.04
Total	233 (100)	19,934 (100)	54.2±11.4	34 (100)	199 (100)	0.2	0.18±0.04

Note: Median, mean and standard deviations or number and percentages as appropriate. LGE, late gadolinium enhancement. Studies that had both LGE and perfusion results were categorized as LGE . For these studies, total gadolinium dose is shown.

**Figure 1:**
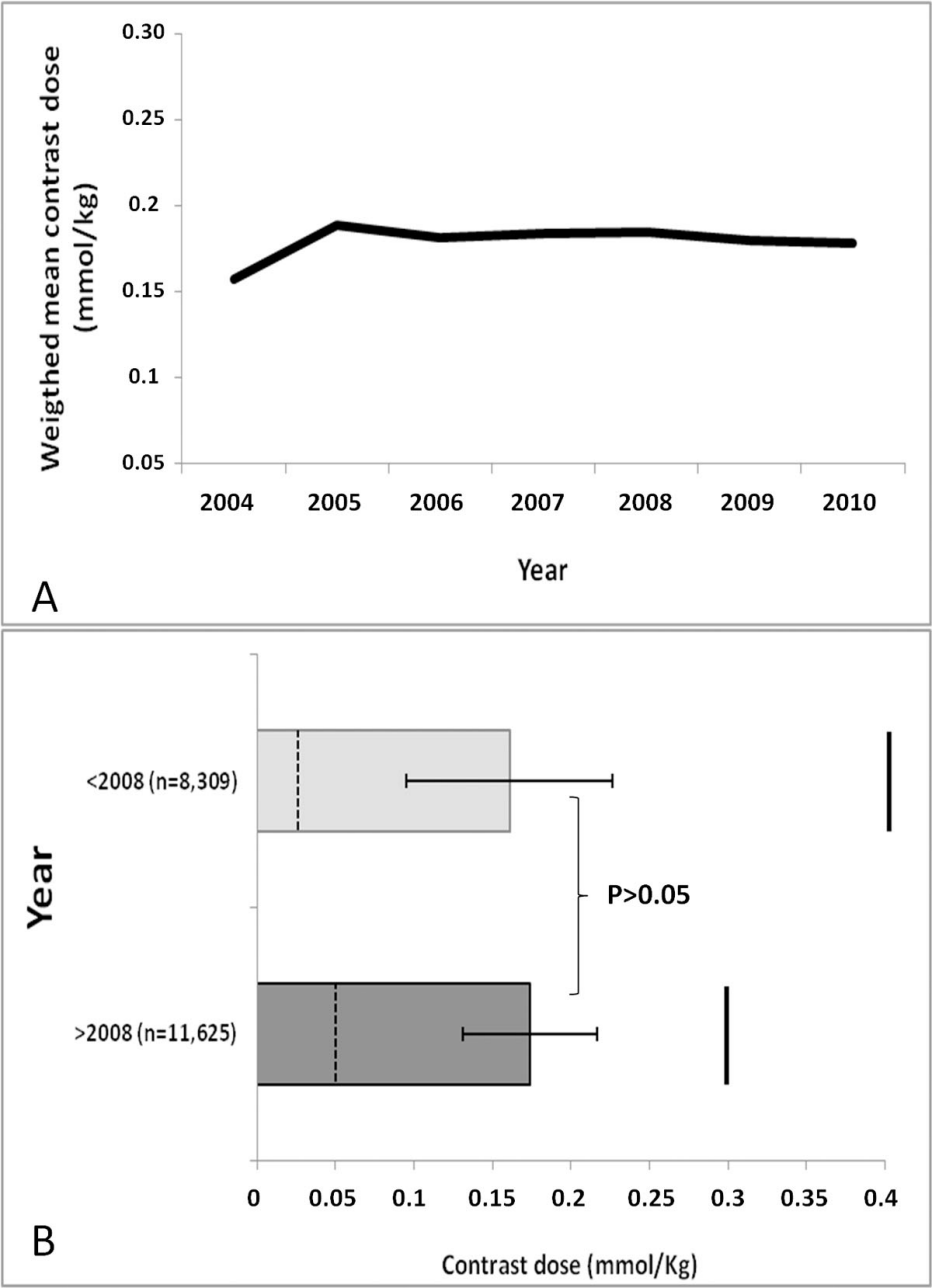
(A) Weighted mean contrast dose (mmol/Kg) from 2004 to 2010. (B) No change in mean gadolinium contrast dose before versus after FDA black box warning. Dashed horizontal lines represents minimum contrast dose and horizontal lines represents maximum contrast dose.

